# Persistent Primary Hyperparathyroidism, Severe Vitamin D Deficiency, and Multiple Pathological Fractures

**DOI:** 10.1155/2016/3016201

**Published:** 2016-07-25

**Authors:** Victoria Mendoza-Zubieta, Mauricio Carvallo-Venegas, Jorge Alberto Vargas-Castilla, Nicolás Ducoing-Sisto, Alfredo Alejandro Páramo-Lovera, Lourdes Josefina Balcázar-Hernández, Julián Malcolm Mac Gregor-Gooch

**Affiliations:** ^1^Endocrinology Department, Hospital de Especialidades, Centro Médico Nacional Siglo XXI, IMSS, 06720 Mexico City, DF, Mexico; ^2^Faculty of Medicine, Universidad Nacional Autónoma de México (UNAM), 04510 Mexico City, DF, Mexico; ^3^Division of Medicine, Hospital de Especialidades, Centro Médico Nacional Siglo XXI, IMSS, 06720 Mexico City, DF, Mexico

## Abstract

Persistent primary hyperparathyroidism (PHPT) refers to the sustained hypercalcemia state detected within the first six months following parathyroidectomy. When it coexists with severe vitamin D deficiency, the effects on bone can be devastating. We report the case of a 56-year-old woman who was sent to this center because of persistent hyperparathyroidism. Her disease had over 3 years of evolution with nephrolithiasis and hip fracture. Parathyroidectomy was performed in her local unit; however, she continued with hypercalcemia, bone pain, and pathological fractures. On admission, the patient was bedridden with multiple deformations by fractures in thoracic and pelvic members. Blood pressure was 100/80, heart rate was 86 per minute, and body mass index was 19 kg/m^2^. Calcium was 14 mg/dL, parathormone 1648 pg/mL, phosphorus 2.3 mg/dL, creatinine 2.4 mg/dL, urea 59 mg/dL, alkaline phosphatase 1580 U/L, and vitamin D 4 ng/mL. She received parenteral treatment of hypercalcemia and replenishment of vitamin D. The second surgical exploration was radioguided by gamma probe. A retroesophageal adenoma of 4 cm was resected.* Conclusion*. Persistent hyperparathyroidism with severe vitamin D deficiency can cause catastrophic skeletal bone softening and fractures.

## 1. Introduction

The classic manifestation of PHPT bone disease is cystic fibrous osteitis, which is clinically characterized by bone pain and radiographically by subperiosteal bone resorption, cysts, and brown tumors [1–3]. The severe deficiency of vitamin D (25-hydroxyvitamin D < 10 ng/dL or <25 nmol/L) produces a lack of mineralization of bone matrix resulting in bone softening and deformities, also called osteomalacia. Persistent PHTP, when coexisting with severe vitamin D deficiency, can have devastating consequences on the skeleton [4–6].

The persistent PHPT is mainly due to delayed diagnosis, lack of localization of ectopic adenomas, and inadequate tumor resection, which prolong the illness and complicate the clinical course [7–10]. First-line treatment and the only one that may be curative in primary hyperparathyroidism (PHPT) is surgery. However, cure rates depend on the experience of the multidisciplinary team; therefore it is important to refer patients to specialized centers with radiologists, surgeons, endocrinologists, and pathologists experienced in the diagnosis and treatment of primary hyperparathyroidism [11–13].

The case presented is a postmenopausal woman with persistent PHPT by ectopic adenoma with multiple atypical fractures due to cystic fibrous osteitis associated with possible osteomalacia.

## 2. Case Report

A 56-year-old woman was referred to a High Specialized Medical Unit with a diagnosis of persistent primary hyperparathyroidism and multiple pathologic fractures.

The patient had no familial history of bone disease or any other chronic-degenerative disease. She had no diabetes, hypertension, or other pathological antecedents. There was no tobacco or alcohol consumption. She presented 3 years earlier with nephrolithiasis and hip fracture due to a fall from her own height that conditioned prostration and limitation of daily living activities. In her local hospital, she fulfilled biochemical criteria for PHPT. No localization studies were performed. She underwent parathyroidectomy with resection of left superior parathyroid and biopsy of left inferior parathyroid; final histopathology reported hyperplasia (the slides of the first histology could not be revised). The surgery was made in another state with surgeons and pathologists inexperienced in parathyroid surgery. The patient persisted with hypercalcemia and elevated levels of PTH during the first 6 months of clinical follow-up; thus, she was sent to our Medical Unit for additional evaluation.

During the first visit to our center, the patient was found with generalized bone pain. On the physical examination, she had normal vital signs including blood pressure 100/80 mmHg, heart rate 86 per minute, and body mass index (BMI) 19 kg/m^2^; she was bedridden and with multiple bone deformities and contractures due to pathologic fractures in thoracic and pelvic extremities.

Laboratory tests reported elevated levels of calcium (14 mg/dL; reference range: 8.4–10.2 mg/dL), low serum phosphorus (2.3 mg/dL; reference range: 2.7–4.5 mg/dL), normal levels of magnesium (2.2 mg/dL; reference range: 1.6–2.6 mg/dL), azotemia with elevated creatinine (2.4 mg/dL; reference range: 0.4–1.2 mg/dL) and urea (59 mg/dL; reference range: 10–50 mg/dL), elevation levels of Intact PTH (iPTH) (1648 pg/mL; reference range: 10–65 pg/mL), severe vitamin D deficiency (<5 ng/mL; sufficiency 30 ng/mL and deficiency < 10 mg/dL), and elevated levels in alkaline phosphatase (1580 UI/L, reference range: 40–129 UI/L) (Table 1). Gonadotropins, thyroid function test, cortisol, and IGF-1 levels were normal for the age of the patient. We corroborated biochemical persistence of PHPT.

**Figure 1:**
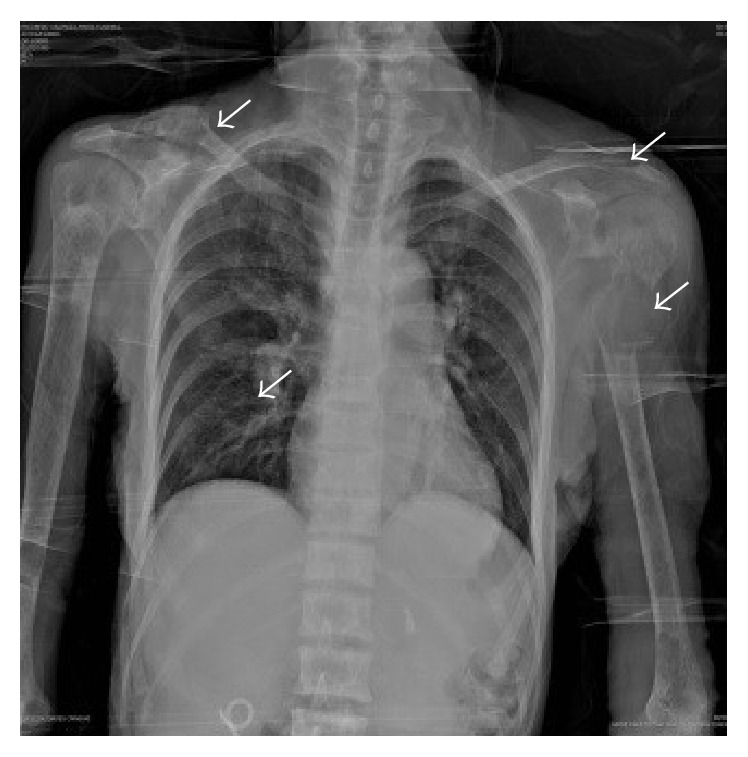
Chest X-ray with severe widespread cortical bone loss, cysts, and brown tumors (arrows) in ribs, distal third of the clavicle, and humerus for severe cystic fibrous osteitis due to prolonged PHPT.

**Figure 2:**
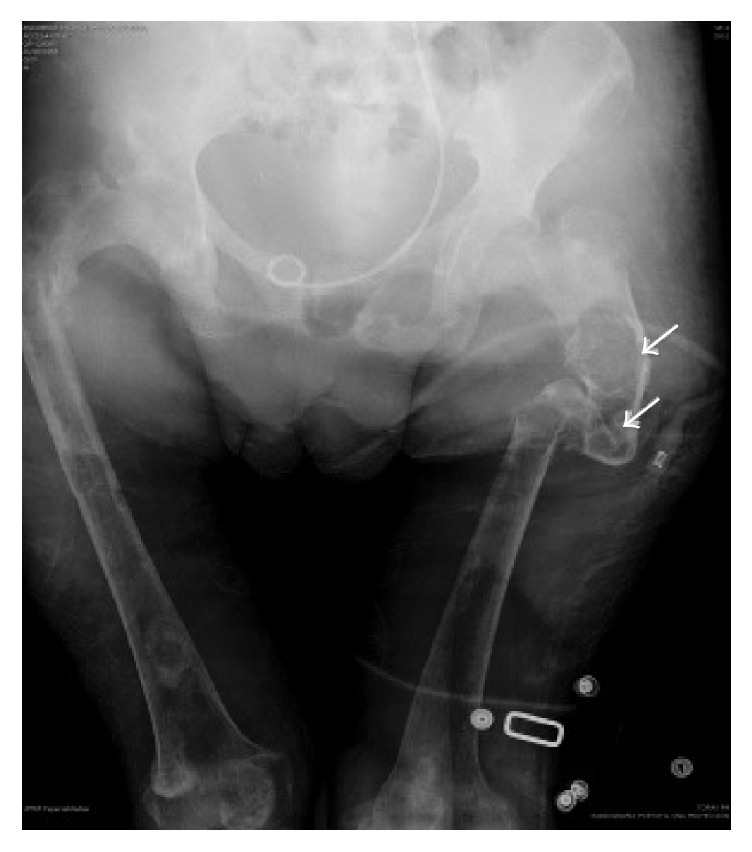
Pelvis X-ray with diffuse demineralization, marked decrease in cortical long bone, cysts, and brown tumors (arrows) and multiple pathological fractures for severe osteitis fibrous cystic.

Radiographically, chest and pelvis X-ray showed severe widespread cortical bone loss, cysts, brown tumors, and multiple pathological fractures because of severe cystic fibrous osteitis (Figures 1 and 2). No localization studies for parathyroid pathologies could not be performed due to the duration of the studies and the pain induced by the position required to perform them.

**Figure 3:**
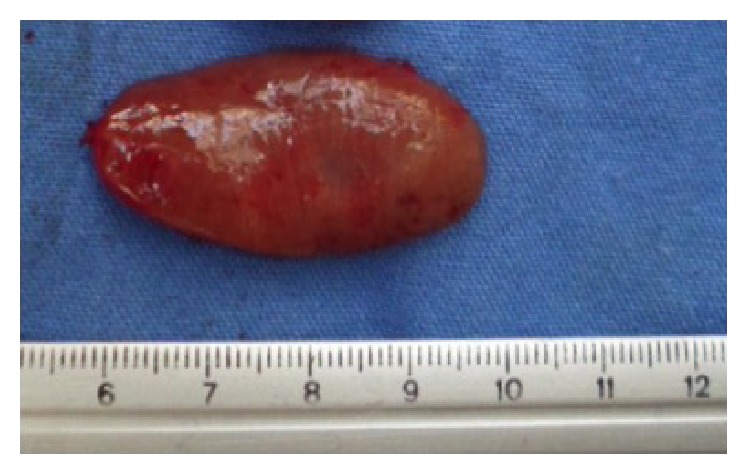
Ectopic parathyroid adenoma with approximately 4 cm dimension in the larger diameter.

We initiated treatment with aggressive intravenous hydration with 0.9% sodium chloride solutions, loop diuretics, and intravenous calcitonin 48 hours before surgery. An experienced head and neck surgeon performed the second surgical bilateral neck exploration with radioguided minimally invasive parathyroidectomy using hand-held gamma probe, evidencing an ectopic adenoma approximately 4 cm in larger diameter (Figure 3).

Postoperatively, the patient presented symptomatic hypocalcemia, with a nadir of 6.8 mg/dL and biochemical evidence of hungry bone syndrome, requiring treatment with intravenous calcium up to 2 mg/kg/min, intravenous magnesium, and oral calcitriol replacement. Despite intensive multidisciplinary approach, the patient had a torpid clinical evolution and died a month later from respiratory complications due to nosocomial pneumonia.

## 3. Discussion

The PHPT is a common endocrine disease with the highest incidence in postmenopausal women. It is characterized by hypercalcemia and elevated levels of PTH [1]. In the last few years, the most common presentation of PHPT has been the asymptomatic form with incidental findings of hypercalcemia in screening laboratories or during the evaluation of patients with nephrolithiasis, osteoporosis, or pathologic fractures [1–3].

Persistent PHPT refers to a sustained hypercalcemia state detected within the first six months following parathyroidectomy, and recurrent PHPT is applicable when the patient has been normocalcemic for at least six months after surgery and then hypercalcemia reappears [8], the most common cause of persistent PHPT in the presence of an ectopic gland that was not identified in the first intervention. One frequent cause of persistent PHPT is surgeon inexperience in locating and adequately excising a parathyroid adenoma. Other causes include a previously unnoticed multiglandular disease (multiple adenomas or hyperplasia) or carcinoma [7–10].

Ectopic adenomas are infrequent; the incidence reported is 5 to 20% cases with a variable localization. The ectopic localization of adenomas is related to abnormal cell migration in the embryogenesis that determines the presence of parathyroid gland from the submandibular region to mediastinum [7–9]. It has also been suggested that, during the development of adenomas, the progressive increase in size and weight of the tumor can produce a gradual descent due to gravity as the parathyroid glands are attached to adjacent tissue by a lax fibroconnective tissue [10]. Udelsman and Donovan described that the most frequent sites of ectopic adenomas are the retroesophageal space, thymus, intrathyroidal, carotid sheath, submandibular region, and the aortic window [14].

The definitive treatment of PHPT is surgery [11, 12]. A frequent factor in the persistence of HTP is the surgeon's lack of experience. The experienced surgeon must have extensive knowledge of the anatomy, embryology, biochemistry, and physiology of parathyroid glands and the pathophysiology of their diseases. In the first surgical neck exploration, when there is a suspicion of ectopic adenoma, the experienced surgeon can find and resect the ectopic tissue in 90% of cases [13]. Thus, the parathyroid surgery must always be made by a multidisciplinary team expert in parathyroid diseases.

In a series of PHPT, the ectopic glands were associated with high calcium levels, larger adenoma, and more frequent cystic fibrous osteitis compared with hyperparathyroidism with eutopic gland location [15].

Bone manifestations of severe PHPT have been increasingly rare due to early detection and treatment. The most emblematic manifestation of PHPT is the cystic fibrous osteitis, which is characterized clinically by bone pain and radiographically by subperiosteal bone resorption principally in the phalanges, distal third of the clavicles, and radiological appearance of “salt and pepper” in the skull. Although bone cysts and brown tumors can occur anywhere in the skeleton, they mainly affect the ribs, humerus, and jaw. Brown tumors result from excessive osteoclast activity and by an accumulation of osteoclasts interspersed with fibrous tissue in an area with poor mineralization. The brown color is due to hemosiderin deposits. Brown tumors can form real tumors with extrinsic destructive compression that can cause pathological fractures in long bones. The fibrous cystic osteitis is very rare in developed countries and mainly occurs in patients with hyperparathyroidism with prolonged evolution or aggressive behavior [2, 6, 16, 17].

Vitamin D deficiency in PHPT patients is related to higher PTH levels, larger adenomas, and severe clinical evolution, including fibrous cystic osteitis and hungry bone syndrome in postsurgery [4, 5, 18]. Vitamin D, through its receptor, has essential actions in the parathyroid cells. It participates in the regulation of PTH secretion and inhibits cell proliferation. The effect of vitamin D deficiency in the pathogenesis of secondary hyperparathyroidism and parathyroid hyperplasia in elderly, renal insufficiency, and intestinal malabsorption syndromes is well established. With the prolonged evolution of PHPT and severe deficiency of vitamin D, adenomas can grow and secrete greater levels of parathyroid hormone causing greater hypercalcemia and greater target organ damage, mainly bone and kidney [2, 4, 5].

The present case report describes a patient with prolonged and severe PHPT with a devastating effect on bone and multiple pathological fractures in hips, pelvic, and thoracic members that conditioned prostration and limitation in daily living activities. Our patient had multiple risk factors like age, prolonged evolution of HPTP, and vitamin D deficiency that contributed to the severe bone affection in a rare presentation of persistent PHPT.

## Figures and Tables

**Table 1:** Laboratory test at hospitalization and follow-up after surgery.

Laboratory test	Reference range	Baseline	8 days after surgery	One month after surgery
Urea (mg/dL)	10.0–50.0 mg/dL	59	93	96
Creatinine (mg/dL)	0.4–1.2 mg/dL	2.4	1.27	1.49
Calcium (mg/dL)	8.4–10.2 mg/dL	14	6.8	8.2
Phosphorus (mg/dL)	2.7–4.5 mg/dL	2.3	2.1	3.5
Magnesium (mg/dL)	1.6–2.6 mg/dL	2.2	1.1	1.8
Alkaline phosphatase (UI/L)	40–129 UI/L	1580	1297	910
PTH (pg/mL)	10–65 pg/mL	1648	37.3	37.6
Vitamin D (ng/mL)	Deficiency < 10 mg/mL Insufficiency 10–30 mg/dL, Sufficiency 30–100 mg/dL Toxicity > 100 mg/dL	4	15	17

PTH, parathyroid hormone.
